# Muscle performance but not biomechanics associate with second knee injury in a matched cohort of athletes who passed functional return‐to‐sport criteria after ACL reconstruction

**DOI:** 10.1002/ksa.70245

**Published:** 2025-12-26

**Authors:** Manuel Angel Romero‐Padron, Neal Weldon, Mazie Atteberry, David Matthew Werner, Tyler Kallman, Matthew Alan Tao, Elizabeth Wellsandt

**Affiliations:** ^1^ Department of Orthopaedic Surgery and Rehabilitation University of Nebraska Medical Center Omaha Nebraska USA; ^2^ Department of Orthopaedic Surgery Texas Tech University Health Sciences Center Lubbock Texas USA; ^3^ College of Medicine University of Nebraska Medical Center Omaha Nebraska USA; ^4^ Department of Physical Therapy, School of Education and Health Sciences University of Dayton Dayton Ohio USA; ^5^ Department of Health and Rehabilitation Sciences, Physical Therapy Program University of Nebraska Medical Center Omaha Nebraska USA

**Keywords:** biomechanical deficits, functional performance, muscle imbalances, sports medicine, sports‐related injuries

## Abstract

**Purpose:**

Athletes remain at risk for a second knee injury after anterior cruciate ligament reconstruction (ACLR), even after passing return‐to‐sport (RTS) testing. While biomechanical asymmetries have been linked to reinjury, it is unclear whether deficits persist in athletes who meet RTS criteria. This study compared muscle performance and biomechanical function between athletes who sustained a second knee injury after passing RTS testing and matched controls without reinjury.

**Methods:**

In this case‐control study, 11 athletes who returned to their preinjury sport within one year after ACLR, passed RTS testing, and subsequently sustained a second knee injury were matched to controls without reinjury. Muscle performance and biomechanical assessments were conducted within two weeks of passing RTS testing, prior to second knee injuries. Muscle performance was assessed using isometric and isokinetic strength testing on an electromechanical dynamometer. Biomechanical function was evaluated during bilateral drop vertical jumps, unilateral drop vertical jumps, and single‐legged hop for distance using three‐dimensional motion analysis with embedded force platforms. Muscle performance and biomechanical function were compared between limb (injured, uninjured) and group (second knee injury, no second knee injury) using 2 × 2 mixed analyses of variance (ANOVA).

**Results:**

Eight athletes reinjured the ipsilateral knee and three injured the contralateral knee, with reinjury occurring on average 15.4 ± 5.0 months after ACLR. Compared with controls, the second injury group showed greater quadriceps rate of torque development symmetry during early isometric contraction (0–100 ms; *p* = 0.008). No other significant differences in muscle performance or biomechanical variables were observed.

**Conclusion:**

Greater and more symmetric quadriceps RTD was associated with second knee injury, while biomechanical measures did not differ between groups. These findings suggest that conventional RTS tests may not adequately detect at‐risk athletes after ACLR, and broader assessment strategies may be needed to guide targeted injury prevention.

**Level of Evidence:**

Level III.

**Abbreviations**
40 ms: 40 ms
ACL: anterior cruciate ligament
ACLR: anterior cruciate ligament reconstruction
ANOVA: analyses of variance
BDVJ: bilateral drop vertical jumps
CONSORT: Consolidated Standards of Reporting Trials
GRF: ground reaction force
HFA: hip flexion angle
HFM: hip flexion moment
IC: initial contact
IKDC: International knee documentation committee subjective function form 2000
KAA: knee abduction angle
KAM: knee abduction moment
KFA: knee flexion angle
KFM: knee flexion moment
KFM: knee flexion moment
KFP: knee flexion power
MS: milliseconds
NIH: National institutes of health
OA: osteoarthritis
PCL: posterior cruciate ligament
pKFM: peak knee flexion moment
PT: physical therapy
RTD: rate of torque development
RTD_0‐100_
: peak isometric quadriceps RTD during the first 100 microseconds
RTD_100‐200_
: peak isometric quadriceps RTD during the second 100 microseconds
RTS: return‐to‐sport
SLH: single leg hop
UDVJ: unilateral drop vertical jumps

## INTRODUCTION

Anterior cruciate ligament (ACL) injuries are common among adolescents and young adults, with a peak incidence of 228 per 100,000 person in females aged 14–18 years and 241 per 100,000 in males aged 19–25 [59]. These elevated rates correspond with peak participation in sports that involve cutting and pivoting movements, which are known to increase the risk of ACL injury [15, 47]. It is estimated that up to 90% of these patients seek out surgical reconstruction with the intent to return‐to‐sport (RTS) at the same level as soon as possible [9]. Unfortunately, there is a wide variation in outcomes. Up to one‐third of young athletes who return to cutting and pivoting sports after an ACL reconstruction (ACLR) suffer a second knee injury within 2 years of surgery, and as few as half of young athletes return to their previous competitive level of sport [1, 40, 54, 64, 68]. Additionally, a second ACL injury has been linked with far worse outcomes compared to the first ACL injury, including twice the risk for having both below normal knee function and developing early knee osteoarthritis (OA) [18]. Given these outcomes, there is a critical need to better understand factors that contribute to reinjury after ACLR that can be targeted in secondary preventative interventions.

Several previous studies have focused on non‐modifiable factors that contribute to the risk of a second ACL injury. Younger age, male sex, increased tibial slope, smaller notch width are risk factors of a second ACL injury [3, 38, 39, 42, 43, 46, 49, 61, 62, 68, 70]. Similarly, previous research has examined modifiable risk factors of ACL re‐injury that can be targeted during post‐operative rehabilitation and used to aid RTS decision‐making. These include participation in high‐risk sports, quadriceps strength, symmetric drop jump ability, knee ROM deficits, subjective knee function, psychological readiness, and biomechanical movement patterns [2, 3, 14, 20, 31, 35, 36, 41, 44, 53, 55, 56, 60, 61, 64, 65, 70]. Although passing a clinical RTS test battery—typically defined as achieving ≥90% symmetry in strength, hop tests, and subjective knee scores—has been shown to reduce the risk of reinjury, it does not eliminate it [17]. For instance, Grindem et al. found an 84% reduction in injury risk when RTS criteria were met, and Kyritsis et al. reported a fourfold increased risk in athletes who did not meet them [20, 35]. Conversely, Paterno et al reported no difference in reinjury risk in a population of young athletes who either passed or failed RTS criterion [55]. However, because this study did not control for RTS decision making, the average time to RTS was just 7 months, at which point only 26% of their patients passed their RTS criterion [55]. Beyond RTS criteria, few studies have identified additional risk factors. Paterno et al. and King et al. linked biomechanical asymmetries during tasks such as drop jumps and unplanned change‐of‐direction movements to higher reinjury risk, but it is unknown if athletes in these cohorts had passed RTS criteria when movement patterns were evaluated [29, 56]. Without focusing on athletes who have met RTS criteria, potential contributors to reinjury cannot be identified that are not already currently accounted for in standard RTS protocols.

Our previous work investigated the extent to which a simple clinic‐based RTS testing battery captures laboratory‐based isokinetic muscle performance and biomechanical function impairments. Briefly, we found that although passing clinic‐based RTS testing was associated with ≥90% limb symmetry and the target isometric quadriceps strength of 3.0 Nm/kg, deficits in quadriceps rate of torque development (RTD) and movement asymmetries still persisted. To advance this work, the purpose of this study was to identify if remaining muscle performance and biomechanical deficits after passing an clinic‐based RTS testing battery associated with the occurrence of a second knee injury. We hypothesised that individuals who had a second knee injury would exhibit greater deficits in quadriceps muscle performance and jumping and hopping biomechanics function during the RTS phase compared to those who do not a second knee injury.

## METHODS

### Study design

This study is a secondary analysis of a prospective cohort study aiming to determine if passing a clinic‐based RTS test battery is equivalent to passing a laboratory‐based RTS test battery and to assess the impact of RTS testing on incidence of a second knee injury. Participants were enroled in the late post‐operative rehabilitation phase when the participant and/or their treating physical therapist anticipated that RTS testing could be passed. For this study, participants returned for testing until they successfully passed an objective, clinic‐based RTS test battery of quadriceps strength, hop tests, and subjective knee function as described below. Although all participants were required to complete the RTS test as part of the study protocol, the rehabilitation protocol before returning to sport and the actual timing of their RTS was not controlled by the research team. That decision was made independently by the patients' treating medical teams, with whom we had no involvement with decision making. Our analysis assessed the effect of muscle performance and biomechanical function on second knee injuries after ACLR. Participants with a second injury during the first year after passing clinic‐based RTS testing were matched by sex, age, meniscus repair status, graft type, and primary sport to athletes in the same cohort who returned to their preinjury level of sport without sustaining a second injury.

### Participants

**Figure 1:**
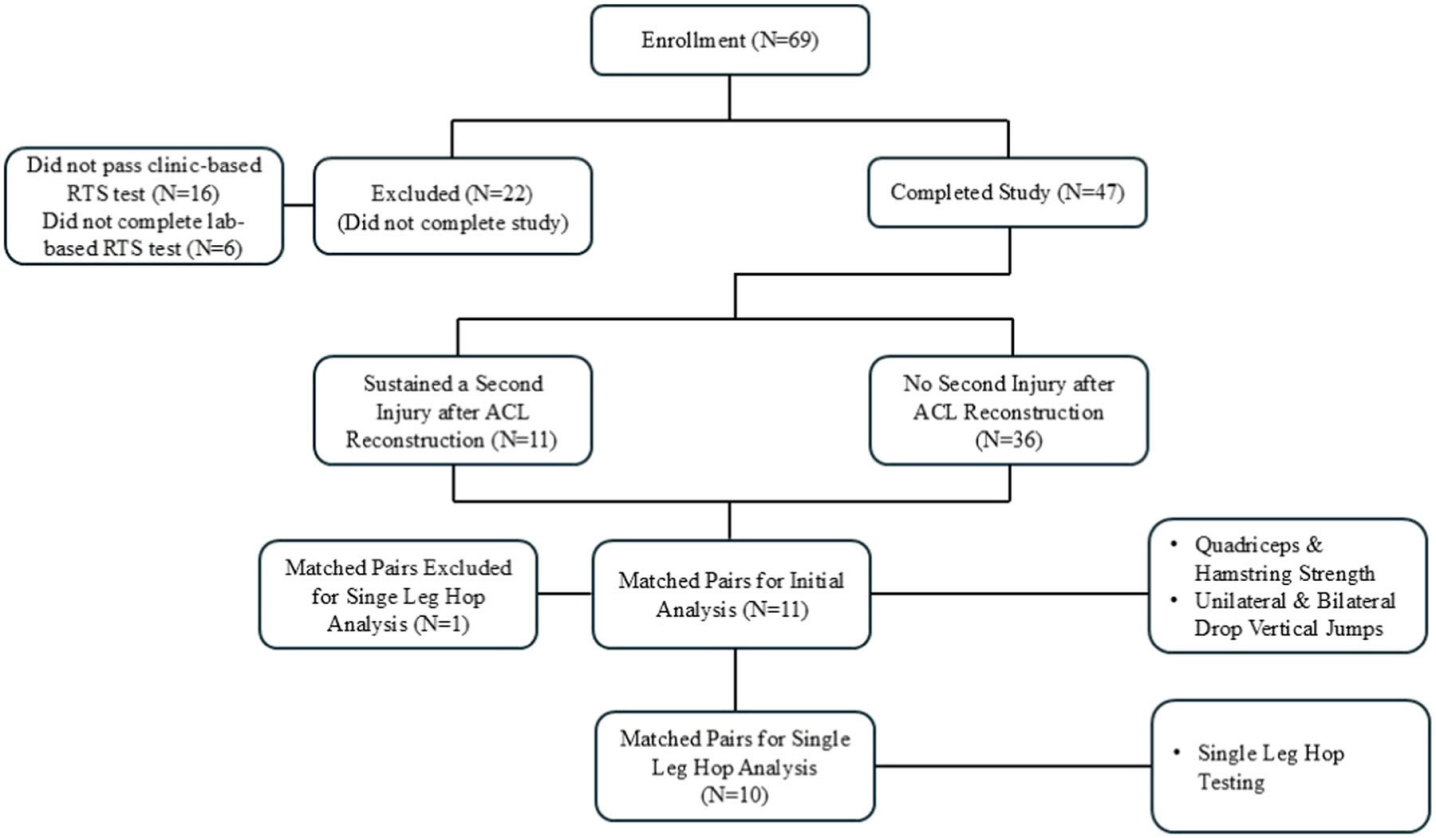
Consolidated Standards of Reporting Trials (CONSORT) Diagram: Participants included in the study. ACL, anterior cruciate ligament; RTS, return‐to‐sport.

Sixty‐nine participants were recruited from local orthopaedic and physical therapy clinics to participate in this prospective cohort study. Participants between the ages of 10–25 who were within 5–15 months of ACLR and were planning to return to at least 50 h per year of Level 1 or 2 cutting and pivoting activities (e.g., basketball and soccer) at the time of enrolment were eligible for the study [12, 19]. Of the 69 original participants, 47 completed all the stages of the study. Exclusion criteria included previous surgery in either knee, concomitant surgical intervention to the posterior cruciate ligament (PCL), current pregnancy, and plans to become pregnant during the study period. All participants provided written informed consent (minors provided assent with written consent from a parent or legal guardian). Eleven athletes who sustained a second injury to either of their knees were matched by sex, graft type, age, sport, competition level, and meniscus repair status to 11 athletes who returned to their preinjury level of sport without sustaining a second injury (Figure 1). This study was approved by the Institutional Review Board at the University of Nebraska Medical Center (IRB# 0215‐20‐EP).

### Participant characteristics and surgical factors

Participants self‐reported age, sex, race, date of ACL injury, duration of post‐operative physical therapy (PT), and pre‐injury level of sport (Level 1‐4) [12, 19]. All study data were collected and/or managed using REDCap electronic data capture tools hosted at the University of Nebraska Medical Center [22, 23]. Operative reports were obtained from each participant's surgeon and were used to identify graft type, date of ACLR, and concomitant meniscal repair status.

### Clinic‐based RTS testing

Before completing laboratory‐based strength and biomechanics testing, participants were required to pass a clinic‐based RTS testing battery of quadriceps strength, single‐legged hop (SLH) test, and patient‐reported knee function. Before testing, participants completed a 5‐min warm‐up on a stationary bike. Physical function measures were completed in the following order: isometric quadriceps strength, isotonic quadriceps strength, and four SLH tests. For strength and hop testing, the uninvolved limb was tested prior to the involved limb. Limb symmetry indices were calculated (involved limb/uninvolved limb × 100% and uninvolved limb/involved limb × 100% for strength tests and hop tests, respectively). Patient‐reported measures were completed in between the isometric and isotonic strength testing to provide a rest break and included the International Knee Documentation Committee Subjective Function Form 2000 (IKDC). To pass RTS testing, participants had to demonstrate at least 90% scores on both quadriceps' strength measures, all four SLH tests, the IKD C, and the GRS. If a participant did not pass the entire RTS test battery, they returned for testing after a time period deemed realistic by the research physical therapist (4–6 weeks) and participant for when passing the RTS test battery could be accomplished. Participants repeated RTS testing until passing all components of the test battery in a single testing session.

#### Quadriceps strength assessment

Quadricep strength was tested in two ways. First, isometric quadriceps strength was measured using a Klau crane scale and a knee extension machine. Participants were secured in the leg extension machine with a non‐extensible strap at the chest and hips and with the pad of the knee extension machine at the thighs. The knee was positioned at a fixed 90‐degree angle using a chain in line with the crane scale and the cushion of the leg extension machine at the distal shin. Participants performed three practice repetitions prior to testing at 50%, 75%, and 100% of maximum effort. Next, three maximal, 5‐s repetitions were completed and recorded in Newtons. At least 60 s of rest was provided between each trial. The best trial (the trial with the greatest demonstrated force output) of each lower extremity was used for analysis. After isometric testing was done, isotonic testing started. A one‐repetition maximum was measured using a knee extension machine to evaluate isotonic quadriceps strength. Participants were secured in the leg extension machine as described above for isometric quadriceps testing. They were instructed to lift the weight from 90° to 0° degrees of knee flexion. With each successful repetition, the resistance was increased in 2.5 to 10‐pound increments until the participant could no longer lift the weight through the full range of motion. At least 60 s of rest was provided between each repetition. The maximum weight that was successfully lifted through the entire range of motion for each lower extremity was recorded in pounds and used for analysis.

#### Single‐legged hop tests

SLH testing consisted of a single hop for distance, crossover hop for distance, triple hop for distance, and 6‐m timed hop, completed in this order [52, 58]. Hop testing is a valid and reliable performance‐based outcome measure following ACLR [52, 58]. Participants practiced each hop test at least once before completing two successful trials for each leg. To be considered successful, the participant had to demonstrate a controlled landing for 2 s by not putting their contralateral foot down, not shifting the foot after landing, and not using the upper extremities for support. Participants rested for at least 30 s between each trial. The average of the two trials in each lower extremity for each hop test was used for analysis.

#### Patient‐reported measure of knee function

Participants completed the IKDC or Pedi‐IKDC and GRS within REDCap data capture following completion of physical testing. The Pedi‐IKDC was administered to participants under age 19, and the IKDC was administered to participants 19 years of age and older. The IKDC is a valid and reliable assessment of physical function after ACLR [26]. The Pedi‐IKDC is a valid and reliable outcome measure of children and adolescents with knee disorders [32]. The GRS, which has also been widely used after ACLR, outlines individual's inability or ability to perform any activity and therefore, the ability to perform at their pre‐injury level of function [25, 27]. The IKDC, Pedi‐IKDC, and GRS are scales scored from 0% to 100%, with values closer to 100% indicating better knee function [24, 26].

### Laboratory‐based testing

Participants who met 90% thresholds on all components of the clinic‐based RTS testing battery during a single session underwent laboratory‐based RTS testing within two weeks of passing clinic‐based RTS testing. Laboratory‐based testing consisted of quadriceps and hamstring strength testing on an isokinetic dynamometer and biomechanical movement analysis, as described below.

#### Quadriceps and hamstring strength assessment

Isometric and isokinetic muscle strength was tested using an electromechanical dynamometer (Biodex System 4 Pro, Shirley, NY) sampled at 100 Hz. Participants were seated in 90° hip flexions with stabilisation straps at the chest, hips, and distal thigh of the limb being tested. The arm of the dynamometer was secured two inches above the lateral malleolus of the tested limb. The uninvolved limb was tested before the involved limb. Participants were instructed to “kick as hard and fast as possible” and given verbal encouragement during each test. Isometric quadriceps strength was tested at 90° knee flexion. Practice trials at 50%, 75%, and 100% effort were completed prior to recorded trials to provide familiarisation to task and ensure absence of pain. Three maximal effort trials lasting five seconds each were completed at each limb. Isokinetic quadriceps and hamstring strength was then tested at a rate of 60°/s from 90° to 0° flexion. Following a practice trial, one trial of five repetitions into alternating concentric knee extension and concentric knee flexion were completed.

**Figure 2:**
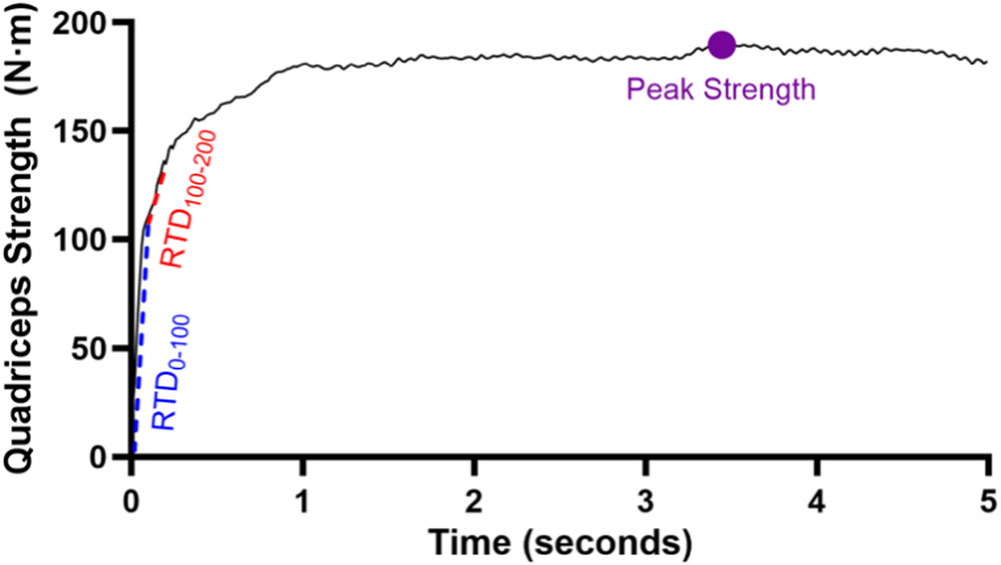
Isometric quadriceps strength curve. Rate of torque development in the first 100 ms (blue dash line), second 100 ms (red dash line) and peak quadriceps strength (purple dot) can be seen in the graph. m, metres; N, newtons; RTD, rate of torque development.

**Figure 3:**
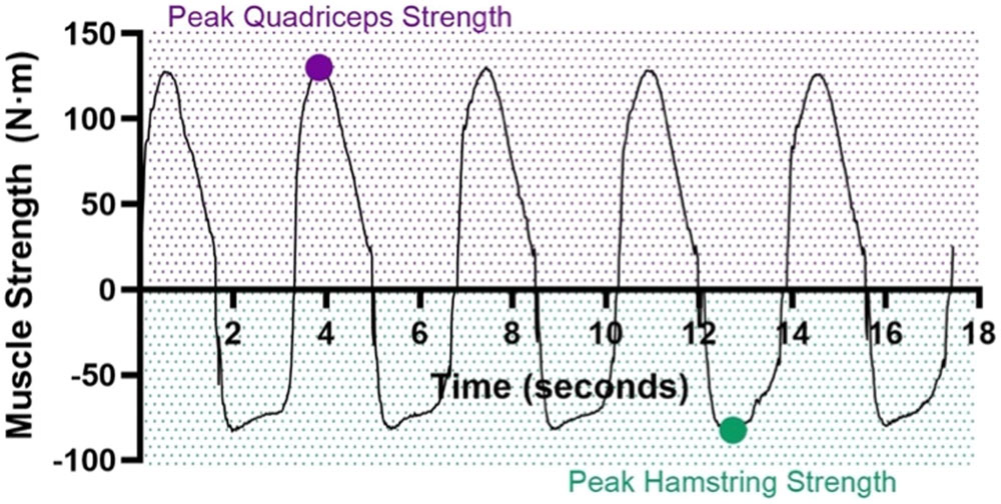
Isokinetic quadriceps and hamstring strength curve. Peak quadriceps (purple dot) and hamstring strength (green dot) can be seen in the graph. m, metres; N, newtons.

Muscle strength data were processed using custom Matlab code (MathWorks, Inc., Natick, MA). Raw data were filtered using a fourth order, low‐pass Butterworth filter with a cut‐off frequency of 8 Hz. Muscle strength variables of interest in each limb included isometric and isokinetic peak quadriceps strength, isokinetic peak hamstring strength, and peak isometric quadriceps RTD during the first 100 ms (ms) (RTD_0‐100_) and the second 100 ms (RTD_100‐200_) (Figures 2 and 3). The best (greatest) of any of the three isometric trials or any of the five isokinetic repetitions in each limb was used for analysis.

#### Biomechanical movement analysis

Kinetic and kinematic data were collected using two embedded force platforms (Bertec Corporation, 2160 Hz; Columbus, OH), a 16‐camera motion capture system (Qualisys Inc, 240 Hz; Goteborg, Sweden) and 29 retroreflective markers with rigid shells (4 markers each) placed at the trunk, pelvis, and lower extremities as previously described [69]. Three foot markers, rigid shells, four pelvis markers, and four trunk markers were used to track the foot, shank/thigh, pelvis, and trunk segments, respectively. Participants completed a one‐second static trial in anatomical position before completing five trials of BDVJ, five trials of UDVJ, and five trials of unilateral hops for distance. For BDVJ and UDVJ, participants stepped off a 30 cm box and each of their feet landed in different force plates (2 plates for BDVJ and 1 plate for UDVJ) [30, 33]. For the SLH trials but before starting recording, participants performed a single‐legged maximal hop in each limb to determine their maximum hopping distance. This measurement was used to adjust the starting position, ensuring they landed on the force platforms. For UDVJ and SLH trials, the uninvolved limb was tested first, followed by the involved limb.

Joint kinematics (angles) and kinetics (moments) were calculated using custom software pipelines developed in our lab (Visual 3D, Has‐Motion; Ontario, Canada). Target and ground reaction force (GRF) data were low‐pass filtered using a fourth‐order bidirectional Butterworth filter with a cutoff frequency of 12 Hz and 50 Hz, respectively. A threshold of 10 N of the vertical GRF was used to define the beginning and end of each limb contact with each force plate [56]. A subject‐specific model was created using height (stadiometer) and mass (static trial), in addition to anatomical marker placement to determine segment lengths and joint centres. Virtual markers at bony landmarks were offset 9 mm toward the bone to account for half of the 14‐mm marker and the 2‐mm base [16]. The ankle and knee joint centres were defined as the mid‐point of the virtual medial and lateral malleoli and virtual medial and lateral femoral epicondyles, respectively. A Visual 3D composite pelvis was built from virtual ASIS and PSIS landmarks. The hip joint center was defined using estimates as previously described by Bell et al. [5, 6] The foot was defined by markers at the first/fifth metatarsal heads and bilateral malleoli, shank by the bilateral malleoli and femoral epicondyles, thigh by the femoral epicondyles and hip joint center, and trunk by center of the pelvis and bilateral acromion. Knee joint moments were calculated using an inverse dynamics approach [71]. Joint moments were normalised to body mass (kg) and height (m) [45]. Values for joint angles, joint moments, and power represent knee flexion, knee flexion and adduction, and knee extension power, respectively.

Variables of interest for UDVJ and BDVJ included knee abduction angle (KAA) at initial contact (IC), knee abduction moment (KAM) at IC, peak knee flexion moment (pKFM) during landing, knee flexion moment (KFM) loading rate during landing and knee flexion power (KFP) during propulsion. For SLH, the variables of interest included hip flexion angle (HFA) at IC and at 40 ms following IC; knee flexion angle (KFA) at IC and 40 ms following IC; hip flexion moment (HFM) at 40 ms following IC; and KFM at 40 ms following IC [56]. Biomechanical variables were analysed at initial contact (IC), based on prior evidence linking this time point to second ACL injury risk, and at their peak values or 40 ms after IC, which reflect knee joint loading during the loading response phase when ACL injuries most commonly occur [4, 34, 56].

### Second knee injuries and return‐to‐sport outcomes

Participants completed an emailed survey using REDCap data capture at 6 and 12 months after completing laboratory‐based RTS testing. This survey included questions about “if” and “when” they had been cleared to RTS. Additional items addressed second injuries, including the presence of a subsequent knee injury, whether it involved the ipsilateral or contralateral knee, the injury mechanism (contact vs. non‐contact), and whether the injury involved the ACL or another knee structure.

### Statistical analysis

All statistical analysis was completed using IBM SPSS Statistics (IBM SPSS Statistics for Windows, Version 30.0. Armonk, NY: IBM Corp). Independent t‐tests and chi‐square tests were used to compare demographics (i.e., age, sex, body mass index (BMI), graft type) as well a time from ACLR to lab testing and time from ACLR to RTS clearance between participants with and without a second knee injury. There were no outliers in the data, as assessed by inspection of a boxplot. Second knee injury status for each level of sex, graft type, age, sport, competition level, and meniscus repair status were normally distributed, and there was homogeneity of variances, as assessed by Levene's test for equality of variances.

Muscle performance and UDVJ, BDVJ, and SLH biomechanics were compared between limb (injured, uninjured) and group (second knee injury, no second knee injury) using 2 × 2 mixed analyses of variance (ANOVA) with post‐hoc Bonferroni analyses to control for multiple between‐ and within‐group comparisons when a significant interaction effect was present. Residual analysis was performed to test the assumptions of the two‐way ANOVA. Outliers were assessed by inspection of a boxplot; normality was assessed by visual inspection using P‐P plots for each cell of the design and homogeneity of variances was assessed by Levene's test. There were no outliers, residuals were normally distributed (*p* > 0.05), and there was homogeneity of variances. 2 × 2 mixed ANOVAs that excluded the two matched pairs of participants with non‐ACL second injuries were also completed and reported in supplemental appendices. Effect sizes for interaction effects (partial *η*²) and within‐ and between‐group comparisons (*d*), as well as 95% CIs for these differences, are reported [11]. This study included multiple comparisons of muscle performance and biomechanical variables between participants with and without a second knee injury. To control for these multiple comparisons, a Benjamini–Hochberg False Discovery Rate correction was applied to the significance levels of the interaction terms within each group of 2 × 2 mixed ANOVA models (i.e., muscle performance, BDVJ, UDVJ, and SLH) [8]. A *p*‐value of 0.05 was set a priori. Sample size for this study was limited to the number of second injuries observed in this cohort. Therefore, a sensitivity analysis was performed in G*Power (version 3.1.9.6) to estimate the minimum detectable effect size. With *α* = 0.05, 80% power, and a sample size of 11 participants per group, the minimum detectable effect size was effect size was 1.26 for independent‐sample comparisons.

## RESULTS

Nine athletes who returned to their preinjury level of sport within the first year after passing RTS testing sustained a second ACL injury to their ipsilateral (*N* = 6) or contralateral (*N* = 3) knee, and two sustained a non‐ACL second knee injury (1 ipsilateral meniscus injury and 1 ipsilateral patellar dislocation). Demographic and baseline characteristics are presented in Table 1.

**Table 1:** Demographic & baseline characteristics between those with and without a second knee injury.

	Number of participants (%) or mean ± SD (95% CI)		*p* value
	Second injury	No second injury	
Age (years)	16.7 ± 2.7 (14.9–18.5)	15.7 ± 2.3 (14.2–17.3)	0.372
Sex (male)	7 (63.6%)	7 (63.6%)	1.00
BMI (kg/m²)	25.2 ± 3.2 (23.0–27.3)	24.1 ± 6.2 (19.9–28.3)	0.631
Autograft type			0.807
Hamstring tendon	4 (36.4%)	5 (45.5%)	
Patellar tendon	2 (18.2%)	3 (27.3%)	
Quadriceps tendon	4 (36.4%)	2 (18.2%)	
Iliotibial band	1 (9.1%)	1 (9.1%)	
Concomitant meniscus repair (yes)	6 (54.5%)	7 (63.6%)	0.665
Type of second knee injury			
Ipsilateral ACL	6 (54.5%)	‐	‐
Contralateral ACL	3 (27.3%)	‐	‐
Non‐ACL reinjury	2 (18.2%)	‐	‐
ACLR to passing clinic‐based RTS testing (months)	10.3 ± 2.8 (8.5–12.2)	9.9 ± 2.2 (8.4–11.4)	0.706
ACLR to returning to preinjury sport level (months)	9.3 ± 2.5 (7.6–11.0)	8.6 ± 1.7 (7.4–9.7)	0.420
ACLR to second knee injury (months)	15.4 ± 5.1 (12.0–18.8)	‐	‐
Time of return to preinjury sport level to second injury (months)	6.0 ± 4.5 (3.0–9.0)	‐	‐

Abbreviations: %, percentage; ACLR, anterior cruciate ligament reconstruction; BMI, body mass index; CI, confidence interval; kg, kilograms; M, male; m, metres; RTS, return‐to‐sport; SD, standard deviation.

**Figure 4:**
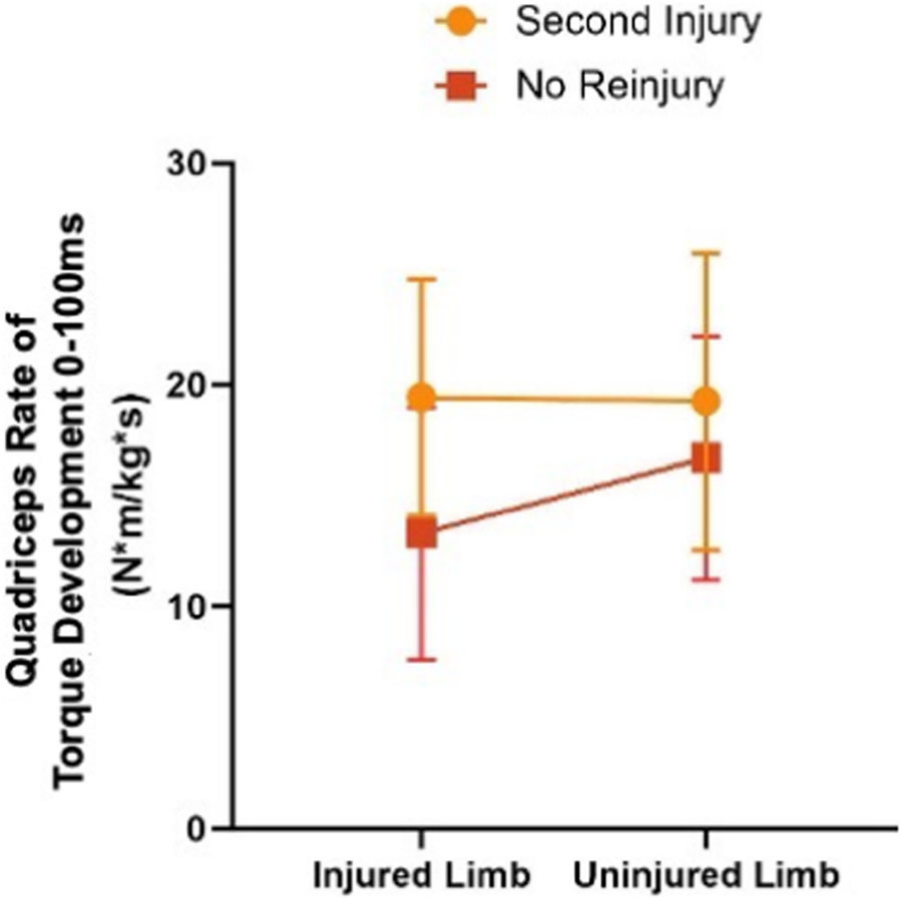
Significant group by limb interaction effects on isometric quadriceps rate of torque development from 0 to 100 ms. kg, kilograms; m, metre; ms, milliseconds; N, newton; Quad, quadriceps; RTD, rate of torque development; s, seconds.

There was a significant limb by group interaction effect for RTD_0‐100_ (*p* = 0.008, partial *η*²: 0.303) (Table 2 and Figure 4). Athletes who sustained a second injury had more symmetric isometric quadriceps RTD_0‐100_ (*p* = 0.854; mean difference: 0.16 N·m/kg·s [95% CI: −1.93 to 2.25 N·m/kg·s], *d*: 0.05 [95% CI: −0.54 to 0.64]) compared to those without a second injury (*p* = 0.001; mean Difference: −3.39 N·m/kg·s [95% CI: −5.07 to −1.71 N·m/kg·s], *d*: −1.36 [95% CI: =2.17 to −0.51]). RTD_0‐100_ in the injured limb was 6.11 N·m/kg·s higher in the second injury group (*p* = 0.017; 95% CI of mean difference: 1.22–11.00 N·m/kg·s, *d*: 1.11 [95% CI: 0.20–2.00]), but there was no significant difference in the uninjured limb (*p* = 0.337; mean difference: 2.57 N·m/kg·s [95% CI: −2.87 to 8.00 N·m/kg·s], *d*: 0.42 [95% CI: −0.43 to 1.26]). While there were no other significant limb by group interaction effects, there were statistically significant main effects of limb for isometric peak quadriceps torque (*p* = 0.005) and isometric quadriceps RTD_100‐200_ (*p* < 0.001) (Table 2), with the original ACL‐injured limb demonstrating lower quadriceps strength and RTD (Table 2).

**Table 2:** Analysis of variance (ANOVA) for quadriceps and hamstring muscle performance outcomes between group and limb.

	Second injury		No second injury		*p* value		
	INJ	UN	INJ	UN	Limb	Group	interaction
Isometric peak quad torque (N·m/kg)	3.49 ± 0.92	3.71 ± 1.14	3.15 ± 0.93	3.48 ± 0.76	0.005*	0.484	0.522
Isokinetic peak quad torque (N·m/kg)	2.31 ± 0.63	2.39 ± 0.72	2.03 ± 0.44	2.19 ± 0.39	0.057	0.327	0.499
Isokinetic peak ham torque (N·m/kg)	1.29 ± 0.45	1.22 ± 0.40	1.16 ± 0.34	1.16 ± 0.27	0.396	0.537	0.400
Isometric quad RTD_0–100 ms_ (N·m/kg·s)	19.42 ± 5.32	19.26 ± 6.69	13.31 ± 5.68	16.70 ± 5.48	0.014*	0.086	0.008**
Isometric quad RTD_100–200 ms_ (N·m/kg·s)	6.69 ± 2.37	9.07 ± 3.21	5.85 ± 2.28	7.03 ± 2.25	<0.001*	0.167	0.173

Abbreviations: Ham, hamstring; INJ, injured limb; kg, kilograms; m, metre; ms, milliseconds; N, newton; Quad, quadriceps; RTD, rate of torque; s, seconds; UN, uninjured limb.

* Represents *p* < 0.05.

** Represents *p* < 0.01 (adjusted).

There were no significant limb by group interaction effects during the BDVJ and UDVJ. (Table 3). Main effects of limb were present for pKFM during landing (BDVJ, *p* < 0.001; UDVJ, *p* = 0.013), KFM loading rate during landing (BDVJ, *p* = 0.044) and KFP during propulsion (BDVJ, *p* < 0.001; UDVJ, *p* < 0.001) with the original ACL‐injured limb always demonstrating lower values than the uninjured limb (Table 3).

**Table 3:** Analysis of variance (ANOVA) for drop vertical jump biomechanics between group and limb.

		Second injury		No second injury		*p* value		
		INJ	UN	INJ	UN	Limb	Group	Interaction
BDVJ	KAA_IC_ (°)	−1.94 ± 3.42	−2.96 ± 5.11	−1.71 ± 3.39	−0.82 ± 3.53	0.950	0.371	0.379
	KAM_IC_ (N·m/kg·m)	0.01 ± 0.03	0.04 ± 0.02	0.02 ± 0.04	0.02 ± 0.03	0.106	0.965	0.079
	pKFM during landing (N·m/kg·m)	0.90 ± 0.15	1.08 ± 0.22	0.84 ± 0.15	1.07 ± 0.20	<0.001*	0.567	0.497
	KFM loading rate during landing (N·m/kg·m·s)	15.78 ± 13.04	12.62 ± 7.36	28.59 ± 26.92	17.50 ± 19.11	0.044*	0.220	0.245
	KFP during propulsion (W/kg·m)	538.65 ± 223.03	615.11 ± 192.27	499.61 ± 241.74	601.74 ± 257.80	<0.001*	0.790	0.377
UDVJ	KAA_IC_ (°)	−0.18 ± 3.54	−0.12 ± 4.84	1.15 ± 2.99	−0.02 ± 3.05	0.515	0.595	0.471
	KAM_IC_ (N·m/kg·m)	0.00 ± 0.03	0.01 ± 0.03	0.00 ± 0.05	0.02 ± 0.04	0.081	0.922	0.916
	pKFM during landing (N·m/kg·m)	1.30 ± 0.38	1.42 ± 0.32	1.31 ± 0.19	1.49 ± 0.14	0.013*	0.727	0.577
	KFM loading rate during landing (N·m/kg·m·s)	14.58 ± 5.45	15.24 ± 5.60	15.97 ± 3.59	17.61 ± 3.02	0.265	0.271	0.630
	KFP during propulsion (W/kg·m)	400.78 ± 189.61	517.96 ± 213.43	355.65 ± 156.83	491.67 ± 221.41	<0.001*	0.666	0.643

Abbreviations: °, degrees; BDVJ, bilateral drop vertical jump; IC, at initial contact; INJ, injured limb; KAA, knee abduction angle; KAM, knee abduction moment; KFM, knee flexion moment; KFP, knee flexion power; kg, kilograms; m, metre; N, newton; pKFM, peak knee flexion moment; s, seconds; UN, uninjured limb; UDVJ, unilateral drop vertical jump; W, watts.

* Represents *p* < 0.05.

There were also no significant interaction effects for any of the SLH variables, but main effects of limb were again present (Table 4). HFA at initial contact (*p* = 0.004) and at 40 ms following IC (*p* = 0.010) were greater in the original ACL‐injured limb compared to the uninjured limb. In addition, KFM at 40 ms following IC was smaller in the original ACL‐injured limb compared to the uninjured limb (*p* = 0.012) (Table 4).

**Table 4:** Analysis of variance (ANOVA) for single‐legged hop biomechanics between group and limb.

		Second injury		No second injury		*p* value		
		INJ	UN	INJ	UN	Limb	Group	Interaction
SLH	KFA_IC_ (°)	9.91 ± 7.03	9.53 ± 6.36	13.00 ± 5.04	11.92 ± 5.47	0.522	0.279	0.759
	KFA_40ms_ (°)	25.28 ± 9.19	24.81 ± 9.19	26.53 ± 6.33	26.24 ± 6.62	0.819	0.674	0.958
	KFM_40ms_ (N·m/kg·m)	0.60 ± 0.39	0.88 ± 0.42	0.71 ± 0.22	0.86 ± 0.24	0.012*	0.703	0.397
	HFA_IC_ (°)	33.74 ± 6.96	31.40 ± 7.16	39.71 ± 9.24	34.12 ± 8.46	0.004*	0.213	0.199
	HFA_40ms_ (°)	42.10 ± 7.73	40.41 ± 7.53	48.06 ± 9.53	41.78 ± 8.64	0.010*	0.307	0.116
	HFM_40ms_ (N·m/kg·m)	1.17 ± 0.83	0.99 ± 0.64	1.23 ± 0.34	0.99 ± 0.50	0.105	0.906	0.838

Abbreviations: °, degrees; HFA, hip flexion angle; HFM, hip flexion moment; IC, initial contact; INJ, injured limb; KFA, knee flexion angle; KFM, knee flexion moment; kg, kilograms; m, metre; ms, milliseconds; N, newton; SLH, single‐legged hop; UN, uninjured limb.

* Represents *p* < 0.05.

Limb by group comparisons of muscle performance and biomechanical variables including only matched pairs of participants with second ACL injuries (*N* = 9) are presented in Appendix Tables 1–3. The only significant limb by group interaction effect was for isometric quadriceps RTD_0‐100_ (*p* = 0.047), consistent with the full sample analysis. The effect size for this subset analysis was similarly large (Cohen's *d* ≈ 1.0, 95% CI: 0.05–1.95).

## DISCUSSION

The purpose of this study was to compare muscle performance and biomechanical function in a matched cohort of young athletes who, after passing a clinic‐based RTS battery after primary ACLR, RTS with or without sustaining a second knee injury. These data failed to support the hypothesis that a second knee injury was associated with remaining deficits in muscle performance during isokinetic and isometric testing as well as biomechanical function during BDVJ, UDVJ and SLH. Although quadriceps strength and biomechanical asymmetries are present in patients with a second injury after ACLR, those alone might not be sufficient predictors of second ACL injury risk, highlighting the need for a more comprehensive approach to RTS assessments and a second knee injury prevention.

### Second knee injury after ACL reconstruction

Previous studies have demonstrated that achieving RTS criteria, including at least 90% quadriceps strength and SLH test symmetry and high levels of subjective knee function, reduces reinjury risk [20, 35]. Quadriceps strength is the most predictive reinjury factor of these included RTS criteria [20]. Our cohort of young individuals returning to high levels of cutting and pivoting sports after ACLR represents the highest risk group for re‐injury [3, 70]. Our study design in the current study is unique compared to previously completed studies, because muscle performance and biomechanical function were assessed only after participants passed current standards for RTS testing (i.e., quadriceps strength, hop tests and subjective knee function). Despite meeting RTS criteria, nearly 20% of individuals in our study sustained a second ACL injury (ipsilateral or contralateral), consistent with prior research reporting second knee injury rates as high as 30% [2, 40, 44, 54, 64, 68]. These data suggest that although commonly used RTS criteria such as quadriceps strength, SLH tests and subjective knee function may represent the minimum thresholds for clearance, more comprehensive assessments are likely required to accurately discriminate between athletes who are adequately prepared for RTS and those who remain at elevated risk for reinjury.

### Muscle performance

Contrary to our hypothesis, participants who sustained a second knee injury exhibited greater isometric quadriceps RTD symmetry compared to those without a second injury. The more symmetric isometric quadriceps RTD was primarily driven by greater RTD in the injured limb of those with a second knee injury. A similar pattern was seen for other quadriceps strength measures that did not exhibit a significant interaction effect but did show a main effect of limb. The implications of this finding remain unclear. One possible explanation is that athletes with greater early RTD may generate higher forces and mechanical demands within their injured knees upon returning to sport, potentially increasing their risk of reinjury. Another possible explanation is that athletes with higher quadriceps RTD symmetry may feel better prepared for RTS and demonstrate athletic performance levels that allow for immediate competitive participation in sport, resulting in an abrupt increase in athletic exposure and opportunities for second knee injury. Previous work further supports the possible link between better levels of ACL recovery and higher reinjury risk. Zarzycki et al. and Paterno et al. found that patients with higher self‐confidence (Knee Injury and Osteoarthritis Outcome Score ‐ Quality of Life Subscale) and psychological readiness (ACL Return‐to‐Sport after Injury Scale) before returning to sport after ACLR have a significantly higher risk of a second knee injury [51, 72]. Prior research has also demonstrated that sudden spikes in training load and exposure are associated with a heightened risk of sports injuries [7, 28, 57, 67].

Beyond symmetry, the faster rate of torque development observed in the reinjured group warrants further consideration. Higher RTD reflects greater neuromuscular explosiveness, which may subject a healing graft and surrounding structures to rapid, high‐intensity loading. Such loading could exceed the tolerance of the biologically immature ACL and previously injured knee. This concept aligns with findings from Simonson et al., who reported that greater quadriceps strength after ACLR was associated with an elevated risk of a second knee injury, although this effect was attenuated when symmetry was accounted for [63]. Importantly, their work focused on overall strength and did not consider RTD, suggesting that explosive strength production, even in the presence of symmetrical recovery, may represent a unique risk factor for reinjury.

Future research is needed to determine if additional RTS criteria are effective in lowering reinjury risk (e.g., documented progression of sport‐related activities), particularly in young athletes returning to competitive cutting and pivoting sports.

### Biomechanical function

Our findings contrast with prior research suggesting that biomechanical patterns are associated with second knee injury after ACLR [29, 56]. The frontal plane hip angle and moment at minimum center of mass during the SLH approached statistically significant limb by group interaction effects. However, for both variables, the second knee injury group demonstrated better symmetry than the group without a second injury. While previous work has linked second knee injury with greater interlimb asymmetries in sagittal and frontal plane biomechanics during BDVJ, our findings did not support this relationship [29, 56]. These previous studies did not control for quadriceps strength. For example, quadriceps strength was not reported by Paterno et al., and only half of individuals in the study by King et al. demonstrated at least 90% quadriceps strength symmetry [29, 56]. In contrast, all participants in our current study demonstrated at least 90% symmetry using clinical measures of quadriceps strength. Quadriceps strength is strongly associated with lower limb biomechanics after ACLR; thus, quadriceps strength may be mediating the relationship between biomechanical function and reinjury risk after ACLR [37, 50]. Additional work by Capin et al. supports our findings that asymmetrical movement patterns may not be consistently predictive of reinjury risk. Capin and colleagues found that individuals with a second knee injury after ACLR and successful RTS had demonstrated more symmetrical knee flexion angles during gait prior to their second injury [10].

### Persistent quadriceps strength and biomechanical asymmetries

All participants passed clinic‐based RTS testing, yet interlimb asymmetries and deficits in quadriceps strength, RTD, and knee joint loading remained. These impairments were present in both those who did and did not sustain a second knee injury. Several previous studies have highlighted the impact of persistent interlimb asymmetries after ACLR, with the most consequential being the development of early‐onset knee osteoarthritis [48, 56]. Further development of low‐cost and feasible tools to identify deficits in muscle performance and movement patterns is needed to reduce gaps between clinic‐based and laboratory‐based testing and ensure optimal patient recovery.

### Strengths and limitations

This is the first study to match participants with and without a second knee injury after ACLR after passing a RTS testing battery of quadriceps strength, SLH tests and patient‐reported outcomes. This study design enabled investigation of additional muscle performance and biomechanical factors of second knee injury following current evidence‐based recommendations for successfully returning to sport after ACLR [1, 13, 56]. In addition, this study included young people returning to high levels of cutting and pivoting sports who are at highest risk for second knee injuries. This study has several limitations. First, the sample size was small, which limited statistical power to detect group differences and interaction effects with smaller but possibly clinically meaningful effects sizes. Second, knee injuries were determined based on self‐report data and only for 1 year following passing of clinic‐based RTS testing. Additional injuries may have occurred that were not included in this analysis. Another possible explanation for some of our findings is that clinical‐based and laboratory‐based biomechanical tests do not fully replicate the real‐world demands of training or competition in cutting and pivoting sports, both in terms of movement patterns and environmental conditions. Moreover, some sports may not heavily incorporate the specific biomechanical tasks analysed in this study, potentially limiting their relevance in predicting second injuries after ACLR. In a motion analysis lab, athletes perform movements in a controlled setting, where they are consciously aware of their actions. However, on the field, these movements must become automatic and instinctive. Therefore, athletes may benefit with the inclusion of neurocognitive training and testing to cover this crucial part of RTS and sports [66]. Additionally, hamstring RTD, a potential ACL agonist, was not included in this analysis. Finally, participants under 19 years of age completed the Pedi‐IKDC; however, recent reports have raised concerns regarding its structural validity, so the corresponding findings should be interpreted with caution [21].

## CONCLUSION

Greater and more symmetric quadriceps RTD was associated with second knee injury, while biomechanical measures did not differ between groups. These findings suggest that conventional RTS tests may not adequately detect at‐risk athletes after ACLR, and broader assessment strategies may be needed to guide targeted injury prevention.

## AUTHOR CONTRIBUTIONS

*Conceptualisation*: Neal Weldon, David Matthew Werner, Tyler Kallman, and Elizabeth Wellsandt. *Methodology*: Manuel Angel Romero‐Padron, Neal Weldon, David Matthew Werner, Mazie Atteberry, and Elizabeth Wellsandt. *Formal Analysis*: Manuel Angel Romero‐Padron and Elizabeth Wellsandt. *Investigation*: Neal Weldon, Mazie Atteberry, David Matthew Werner, Tyler Kallman, and Elizabeth Wellsandt. *Resources*: Manuel Angel Romero‐Padron, Neal Weldon, David Matthew Werner, and Elizabeth Wellsandt. *Data Curation*: Manuel Angel Romero‐Padron, Neal Weldon, David Matthew Werner, and Elizabeth Wellsandt. *Writing–Original Draft*: Manuel Angel Romero‐Padron, Neal Weldon, and Elizabeth Wellsandt. *Writing –Review and Editing*: Manuel Angel Romero‐Padron, Neal Weldon, Mazie Atteberry, David Matthew Werner, Tyler Kallman, Matthew Alan Tao, and Elizabeth Wellsandt. *Visualisation*: Manuel Angel Romero‐Padron, Neal Weldon, Tyler Kallman. *Supervision*: Matthew Alan Tao and Elizabeth Wellsandt. *Project Administration*: Matthew Alan Tao and Elizabeth Wellsandt. *Funding Acquisition*: Matthew Alan Tao and Elizabeth Wellsandt.

## CONFLICT OF INTEREST STATEMENT

The authors have no professional relationships with companies or manufacturers who will benefit from the results of the present study. EW is funded by the National Institutes of Health (U54GM115458, R01AR080346, R15AG085105) and the Arthritis Foundation (Osteoarthritis Clinical Trials Network (OACTN). MT serves on the editorial board for Current Review in Musculoskeletal Medicine, provides consulting for NewClip and Vericel, and owns stock in Overture and NeAT Surgical.

## ETHICS STATEMENT

This study was approved by the Institutional Review Board at the University of Nebraska Medical Center (IRB#215‐20‐EP). All participants provided written informed consent (minors provided assent with written consent from a parent or legal guardian).

## Supporting information

Supporting information.

## Data Availability

The data that support the findings of this study are available from the corresponding author upon reasonable request.
