# Supplementary material for: Muscle performance but not biomechanics associate with second knee injury in a matched cohort of athletes who passed functional return‐to‐sport criteria after ACL reconstruction
Source: Knee Surg Sports Traumatol Arthrosc. 2025 Dec 26;34(3):1118–30. doi: 10.1002/ksa.70245 (PMC12948342; doi:10.1002/ksa.70245)
Supplement: Supplementary file 1 — Supporting information. [file KSA-34-1118-s001.docx]

| **Appendix Table 1**. Analysis of variance (ANOVA) for quadriceps and hamstring muscle performance outcomes between group and limb. | | | | | | | |
| --- | --- | --- | --- | --- | --- | --- | --- |
|  | Second Injury | | No Second Injury | | p-value | | |
|  | INJ | UN | INJ | UN | Limb | Group | Interaction |
| Isometric Peak Quad Torque (N·m/kg) | 3.69 ± 0.90 | 3.91 ± 1.16 | 3.35 ± 0.90 | 3.61 ± 0.76 | 0.033* | 0.469 | 0.844 |
| Isokinetic Peak Quad Torque (N·m /kg) | 2.34 ± 0.68 | 2.38 ± 0.81 | 2.02 ± 0.45 | 2.18 ± 0.44 | 0.123 | 0.369 | 0.384 |
| Isokinetic Peak Ham Torque (N·m /kg) | 1.32 ± 0.49 | 1.21 ± 0.44 | 1.18 ± 0.35 | 1.14 ± 0.30 | 0.067 | 0.576 | 0.390 |
| Isometric Quad RTD_0-100ms_ (N·m/kg·s) | 19.62 ± 5.92 | 19.55 ± 7.44 | 13.59 ± 5.58 | 16.60 ± 5.70 | 0.057 | 0.133 | 0.047* |
| Isometric Quad RTD_100-200ms_ (N·m/kg·s) | 7.23 ± 2.19 | 9.79 ± 3.02 | 6.33 ± 2.23 | 7.28 ± 2.26 | 0.003* | 0.123 | 0.119 |

*Represents p<0.05.

Abbreviations: INJ: injured limb; UN: uninjured limb; Quad, quadriceps; Ham, hamstring; RTD, rate of torque; N, newton; m, meter; kg, kilograms; s, seconds.

| **Appendix Table 2.** Analysis of variance (ANOVA) for drop vertical jump biomechanics between group and limb. | | | | | | | | |
| --- | --- | --- | --- | --- | --- | --- | --- | --- |
|  |  | Second Injury | | No Second Injury | | p-Value | | |
|  |  | INJ | UN | INJ | UN | Limb | Group | Interaction |
| BDVJ | KAA_IC_ (°) | -2.63 ± 3.29 | -3.50 ± 5.24 | -1.67 ± 3.67 | -1.23 ± 3.59 | 0.863 | 0.279 | 0.602 |
|  | KAM_IC_ (N·m/kg·m) | 0.00 ± 0.03 | 0.04 ± 0.03 | 0.02 ± 0.04 | 0.02 ± 0.03 | 0.089 | 0.841 | 0.056 |
|  | pKFM during landing (N·m/kg·m) | 0.92 ± 0.16 | 1.08 ± 0.24 | 0.85 ± 0.16 | 1.08 ± 0.22 | <0.001* | 0.699 | 0.512 |
|  | KFM loading rate during landing (N·m/kg·m·s) | 15.91 ± 14.15 | 12.73 ± 8.23 | 27.51 ± 26.59 | 17.94 ± 21.33 | 0.062 | 0.328 | 0.330 |
|  | KFP during propulsion (W/kg·m) | 599.56 ± 196.31 | 674.49 ± 153.18 | 537.05 ± 247.93 | 637.18 ± 268.68 | <0.001* | 0.634 | 0.482 |
| UDVJ | KAA_IC_ (°) | -0.99 ± 3.33 | -048 ± 5.24 | 1.26 ± 3.33 | -0.40 ± 3.07 | 0.567 | 0.456 | 0.286 |
|  | KAM_IC_ (N·m/kg·m) | 0.00 ± 0.04 | 0.01 ± 0.03 | -0.01 ± 0.05 | 0.02 ± 0.05 | 0.110 | 0.927 | 0.561 |
|  | pKFM during landing (N·m/kg·m) | 1.27 ± 0.34 | 1.39 ± 0.32 | 1.33 ± 0.19 | 1.49 ± 0.15 | 0.047* | 0.462 | 0.786 |
|  | KFM loading rate during landing (N·m/kg·m·s) | 14.10 ± 5.72 | 14.80 ± 6.09 | 15.92 ± 3.58 | 17.06 ± 3.08 | 0.451 | 0.305 | 0.857 |
|  | KFP during propulsion (W/kg·m) | 442.83 ± 174.07 | 558.97 ± 211.45 | 372.77 ± 161.86 | 529.99 ± 226.16 | <0.001* | 0.586 | 0.379 |

*Represents p<0.05.

Abbreviations: INJ: injured limb; UN: uninjured limb; BDVJ: bilateral drop vertical jump; UDVJ: unilateral drop vertical jump; KAA: knee abduction angle; KAM: knee abduction moment; pKFM: peak knee flexion moment; KFM: knee flexion moment; KFP: knee flexion power; IC, at initial contact; °, degrees; N, newton; m, meter; kg, kilograms; s, seconds; W, watts.

| **Appendix Table 3.** Analysis of variance (ANOVA) for single leg hop biomechanics between group and limb. | | | | | | | | |
| --- | --- | --- | --- | --- | --- | --- | --- | --- |
|  |  | Second Injury | | No Second Injury | | p-Value | | |
|  |  | INJ | UN | INJ | UN | Limb | Group | Interaction |
| SLH | KFA_IC_ (°) | 8.26 ± 5.93 | 8.14 ± 5.67 | 12.81 ± 5.70 | 10.85 ± 5.59 | 0.454 | 0.172 | 0.504 |
|  | KFA_40ms_ (°) | 23.97 ± 9.41 | 23.10 ± 8.69 | 25.86 ± 6.74 | 25.01 ± 6.49 | 0.682 | 0.585 | 0.998 |
|  | KFM_40ms_ (N·m/kg·m) | 0.59 ± 0.44 | 0.82 ± 0.44 | 0.69 ± 0.24 | 0.81 ± 0.24 | 0.091 | 0.773 | 0.568 |
|  | HFA_IC_ (°) | 32.88 ± 7.24 | 30.82 ± 7.67 | 42.16 ± 8.49 | 35.41 ± 9.01 | 0.009* | 0.090 | 0.130 |
|  | HFA_40ms_ (°) | 40.96 ± 8.30 | 40.41 ± 8.34 | 51.15 ± 7.85 | 43.71 ± 8.57 | 0.026* | 0.098 | 0.051 |
|  | HFM_40ms_ (N·m/kg·m) | 0.98 ± 0.78 | 0.87 ± 0.58 | 1.30 ± 0.33 | 0.99 ± 0.56 | 0.194 | 0.419 | 0.479 |

Note: Analysis includes 8 matched pairs of participants due to missing single hop data for one participant.

*Represents p<0.05.

Abbreviations: INJ: injured limb; UN: uninjured limb; SLH, single leg hop; HFA, hip flexion angle; KFA, knee flexion angle; HFM, hip flexion moment; KFM, knee flexion moment; IC, initial contact; minCOM, minimal center of mass; °, degrees; N, newton; m, meter; kg, kilograms.
